# THE INTERSECTIONAL IMPACT OF RACE AND GENDER ON END OF LIFE

**DOI:** 10.1093/geroni/igad104.0803

**Published:** 2023-12-21

**Authors:** Zainab Suntai, Hyunjin Noh, Lewis Lee, Gregg Bell, Megan Lippe, Hee Yun Lee

**Affiliations:** Baylor University, Waco, Texas, United States; University of Alabama, Tuscaloosa, Alabama, United States; School of Social Work, The University of Alabama, Tuscalossa, Alabama, United States; The University of Alabama, Tuscaloosa, Alabama, United States; University of Texas Health Science Center, San Antonio, Texas, United States; The University of Alabama, Tuscaloosa, Alabama, United States

## Abstract

Over the past few decades, research on quality of life at the end of life has proliferated, with an increased focus on issues of diversity. However, few studies have considered the impact of multiple disadvantaged identities, including the combination of race and gender. This study assessed the intersectional impact of race and gender on four end-of-life outcomes: pain, anxiety/depression, patient autonomy, and overall care quality. Data were derived from the combined 2012 to 2020 last-month-of-life interviews conducted by the National Health and Aging Trends, which is an annual longitudinal panel survey of Medicare beneficiaries aged 65 and older. Multivariate logistic regression models were used to test the association between the race/gender intersection and each of the four outcomes. Results showed that Black women were the most likely to be in pain, most likely to have a lack of autonomy in decision-making, and the least likely to have excellent or good care at the end of life. White women were the most likely to have had anxiety/depression, followed by Black women, Black men, and then White men. Across all four outcomes, White men were the most likely to have had favorable outcomes, confirming the theory of intersectionality. These results point to a significant disparity in quality end-of-life care for Black women, who have double-jeopardy as a result of their membership in two vulnerable groups. Thus, there is increased need for practice, policy, and research interventions to attain equitable end-of-life care for all individuals.

